# *Toxoplasma* Infection Induces Sustained Up-Regulation of Complement Factor B and C5a Receptor in the Mouse Brain *via* Microglial Activation: Implication for the Alternative Complement Pathway Activation and Anaphylatoxin Signaling in Cerebral Toxoplasmosis

**DOI:** 10.3389/fimmu.2020.603924

**Published:** 2021-02-05

**Authors:** Noriko Shinjyo, Kenji Hikosaka, Yasutoshi Kido, Hiroki Yoshida, Kazumi Norose

**Affiliations:** ^1^Department of Infection and Host Defense, Graduate School of Medicine, Chiba University, Chiba, Japan; ^2^School of Tropical Medicine and Global Health, Nagasaki University, Nagasaki, Japan; ^3^Department of Parasitology & Research Center for Infectious Disease Sciences, Graduate School of Medicine, Osaka City University, Osaka, Japan; ^4^Division of Molecular and Cellular Immunoscience, Department of Biomolecular Sciences, Faculty of Medicine, Saga University, Saga, Japan

**Keywords:** complement, *Toxoplasma*, cerebral toxoplasmosis, infection, brain, microglia

## Abstract

*Toxoplasma gondii* is a neurotropic protozoan parasite, which is linked to neurological manifestations in immunocompromised individuals as well as severe neurodevelopmental sequelae in congenital toxoplasmosis. While the complement system is the first line of host defense that plays a significant role in the prevention of parasite dissemination, *Toxoplasma* artfully evades complement-mediated clearance *via* recruiting complement regulatory proteins to their surface. On the other hand, the details of *Toxoplasma* and the complement system interaction in the brain parenchyma remain elusive. In this study, infection-induced changes in the mRNA levels of complement components were analyzed by quantitative PCR using a murine *Toxoplasma* infection model *in vivo* and primary glial cells *in vitro*. In addition to the core components C3 and C1q, anaphylatoxin C3a and C5a receptors (C3aR and C5aR1), as well as alternative complement pathway components properdin (CFP) and factor B (CFB), were significantly upregulated 2 weeks after inoculation. Two months post-infection, CFB, C3, C3aR, and C5aR1 expression remained higher than in controls, while CFP upregulation was transient. Furthermore, *Toxoplasma* infection induced significant increase in CFP, CFB, C3, and C5aR1 in mixed glial culture, which was abrogated when microglial activation was inhibited by pre-treatment with minocycline. This study sheds new light on the roles for the complement system in the brain parenchyma during *Toxoplasma* infection, which may lead to the development of novel therapeutic approaches to *Toxoplasma* infection-induced neurological disorders.

## Introduction

*Toxoplasma gondii* is a highly prevalent neurotropic parasite, infecting over one third of the global population. In healthy individuals, it is typically asymptomatic or presents with only mild symptoms such as malaise and fever, which are self-resolving (1). However, it can cause severe diseases, particularly in the retina and brain (cerebral toxoplasmosis), in immunocompromised individuals and children born to mothers exposed to primary infection during pregnancy (2). Vertical transmission of *Toxoplasma* increases the risk of premature birth and stillbirth (3, 4). In addition, it has a significant impact on the fetal brain development, resulting in congenital toxoplasmosis with devastating neurological impairments, including blindness, retinochoroiditis, seizures, and increased lifetime risk for mental illnesses, such as schizophrenia, autism, bipolar disorder, and depression, that may develop later in life (2, 3, 5). Humans and other vertebrates, including rodents, become infected *via* oral ingestion of oocysts shed by the definitive host (felines) or cysts in the tissue of infected animals. After release into the intestinal epithelium, those parasites transform into fast-replicating tachyzoites, the tissue-damaging life stage of *T. gondii*. Due to the immune surveillance, the tachyzoites may transform into slow replicating bradyzoites, the cyst-forming dormant life stage. Cysts are preferentially formed in the brain and retina (6), leading to chronic infection. Coordinated responses of innate and acquired immune systems are crucial in the host defense against *Toxoplasma* infection. By controlling the proliferation of tissue-damaging tachyzoites *via* recruitment of immune cells and molecules, cytokines, including interleukin 12 (IL-12) and interferon γ (IFN-γ), play a central role in the suppression of acute toxoplasmosis (7).

The complement system is the first line of host defense against infection, and can be initiated *via* three major pathways: the classical, lectin, and alternative pathways (8). Recognition of antigen-antibody complexes activates the classical pathway, whereas the lectin pathway is initiated upon mannose-binding lectin (MBL) binding to microbial carbohydrates. The alternative pathway is constantly and spontaneously activated by the hydrolysis of C3 and recognition of foreign surfaces, and is strictly controlled by regulatory proteins, including positive regulators (factor P, also called properdin, and factor B) and a negative regulator (factor H), to prevent potential damage to the host. The recognition triggers a proteolytic cascade that eventually leads to the assembly of the terminal complement complex called the membrane attack complex (MAC), composed of C5b, C6, C7, C8, and several C9 molecules, which disrupts the membrane of the target. In addition, the proteolytic cascade generates anaphylatoxins C3a and C5a, which further enhance innate and adaptive immunity *via* their cognate receptors: C3a receptor (C3aR) and C5a receptors (C5aR1 and C5aR2) (8). C3aR and C5aR1 are expressed both in the murine and human brains, whereas it is unknown whether C5aR2 is expressed in the human brain (9). While the complement system plays an essential role as the first line of defense as well as a bridge between innate and adaptive immunity upon infection, excessive complement activation contributes to a wide range of inflammatory disorders and damages tissues (10, 11).

Although it was originally thought to be involved in innate immunity in the blood, complement components, including C1q, C3, and their receptors are locally produced in the brain parenchyma (12, 13). Growing evidence suggests diverse roles for the complement system in the maintenance of neural circuits during development, aging, and neurological disorders (14, 15). C1q and C3 are critical mediators of synaptic plasticity during development (16, 17), while aberrant activation of these factors is likely involved in neuroinflammation in Alzheimer’s disease (18). C3a plays crucial roles in the regulation of neurogenesis, and protects the developing and mature brain against ischemic injuries (19–21). On the other hand, C5aR1 signaling is associated with poor outcome after subarachnoid hemorrhage in human patients as well as in rodent models (22), and C5a-dependent chemotactic activity is linked to brain damage and disease severity in bacterial meningitis (23). In experimental malaria models, *in utero* exposure-induced cognitive deficits in the offspring are mediated by C5aR1 signaling (24), suggesting detrimental consequences of excessive C5aR1 signaling activity on CNS development. CFB and properdin levels significantly increased in the brain of scrapie-infected mice (25), and alternative complement pathway inhibition improved repair and regeneration after cerebral ischemia and reperfusion injury (26), suggesting that the alternative pathway is activated in response to infection and injury, with detrimental consequences to CNS functionality. Meanwhile, evidence suggests that glial cells produce complement components upon stimulation. Astrocytes constitutively produce C3 (27), the expression of which is enhanced upon stimulation by inflammatory mediators, such as IFN-γ (28). CFB is synthesized in astrocytes in response to stimulation by LPS and IFN-γ (27, 29). Alternative pathway activation is involved in microglial priming and overactivation, which may confer susceptibility to neurodegeneration (30). In addition, C3aR signaling mediates microglia-astrocyte crosstalk and induces microglial polarization (31) as well as chemotaxis (32, 33). Considering the crucial roles for astrocytes and microglia in CNS development and repair after injuries (34, 35), these data suggest the involvement of glia-dependent complement activation in brain development and repair processes.

Although there is no doubt that the complement system is involved in the host defense in the acute phase of *Toxoplasma* infection (36–41), it is only recently that the interaction between *Toxoplasma* and the complement system in the brain has been brought to light. Persistent *Toxoplasma* infection led to the upregulation of C1q, C1r, C3 and C4 and deposition of complement component proteins (C1q and C3) in the brain (42–44), which may be associated with neurodegeneration (43). However, it is uncertain which pathways are persistently activated in the brain parenchyma, and what cell types are responsible for *Toxoplasma* infection-induced complement activation. *Toxoplasma* readily infects both glia (astrocytes and microglia) and neurons, the major cellular components of the brain parenchyma (45). Although cyst formation primarily occurs in neurons (46, 47), interactions between *Toxoplasma* and glial cells play a crucial role during initial parasite dissemination and reactivation (48–50), potentially contributing to the development of encephalitis. In this study, *Toxoplasma* infection-induced changes in the mRNA levels of complement components in the brain and glial cells were examined, focusing on the alternative pathway and anaphylatoxin C3a and C5a receptors, using *in vivo* and *in vitro* infection models.

## Methods

### Reagents

Minocycline hydrochloride was purchased from FUJIFILM Wako Pure Chemical Co. (Osaka, Japan). Sulfadiazine and pyrimethamine were purchased from Tokyo Chemical Industry Co., Ltd. (Tokyo, Japan).

### Animals

C57BL/6J mice were purchased from Japan SLC (Shizuoka, Japan). All animals were housed under specific pathogen-free conditions at Chiba University Animal Facility. This study was carried out according to protocols approved by the Ethics Committee of Chiba University (permit number: A30-149).

### *Toxoplasma* Infection *In Vivo*

Avirulent *T. gondii* Fukaya strain (type II) was used for *in vivo* experiments. C57BL/6J mice (male, 5-week old) were orally infected with three *T. gondii* Fukaya cysts using a syringe fitted with a rounded needle. The brain homogenate of C57/B6J mice 6 weeks post-infection was used as a source of *T. gondii* Fukaya cysts. Mock-infected (control) mice received 200 µL phosphate buffered saline (PBS). Sulfadiazine (200 mg/kg per day) and pyrimethamine (12.5 mg/kg per day) were suspended in water containing 1% carboxymethyl cellulose and orally administered using disposable, flexible feeding needles (Fuchigami Ltd., Kyoto, Japan), from day 1 to day 14 after inoculation. Two or 8 weeks after inoculation, mice were anesthetized with sodium pentobarbital 80 mg/kg (i.p.), and then sacrificed by cervical dislocation. Cerebral cortices were isolated in ice-cold PBS under a stereoscopic microscope. The left and right cortices were used for RNA isolation and DNA extraction, respectively.

### Glial Cell Cultures

Cortical glial cells were prepared from postnatal days 1–2 mice (C57BL/6J) as described previously (51). Briefly, cerebral cortices were isolated in Hank’s Balanced Salt Solution, freed from meninges, and mechanically dissociated using a 70 µm cell strainer (BD Falcon, Durham, NC, USA). Cells were cultured in Dulbecco’s Modified Eagle’s Medium (DMEM; Sigma-Aldrich, St Louis, MO, USA) supplemented with 10% (v/v) fetal bovine serum (FBS; Life Technologies, Bleiswijk, the Netherlands), 2 mM glutamine, and antibiotics (penicillin and streptomycin) at 5% CO_2_ and 37°C. Cells were dissociated using Accutase (Nacalai tesque, Inc. Kyoto, Japan) and re-plated in DMEM containing 10% (v/v) FBS onto poly-D-lysine-coated plates at a density of 20,000 cells/cm^2^. For immunofluorescence, cells were seeded onto poly-D-lysine-coated glass coverslips at the same density. Microglia-enriched culture was prepared by replacing the medium at confluency with DMEM: Nutrient Mixture F-12 (DMEM/F12; Life Technologies) supplemented with 10% (v/v) FBS and antibiotics. Microglia-inactivated cultures were prepared by pre-treatment with 20 µM minocycline, a microglia inhibitor (52, 53), for 5 days prior to use. Medium was replaced with DMEM/F12 containing 2% (v/v) FBS for *T. gondii* infection.

### *Toxoplasma* Infection *In Vitro*

*T. gondii* PTG-GFP strain (type II) (ATCC 50941, ATCC, VA, USA) was amplified in Vero cells and tachyzoites were isolated for *in vitro* assays as described previously (54). Isolated tachyzoites were seeded onto glial cells at 10,000 tachyzoites/cm^2^ in DMEM/F12 containing 2% (v/v) FBS, with or without anti-*Toxoplasma* drug pyrimethamine (2 µM), and incubated for 48 h prior to DNA or RNA extraction.

### Quantitative Real-Time PCR

RNA isolation was performed using RNAiso Plus (TAKARA BIO INC., Shiga, Japan) according to the manufacturer’s instruction. Reverse transcription was performed using ReverTra Ace qPCR RT Master Mix with gDNA Remover (TOYOBO Co., Osaka, Japan). Genomic DNA (gDNA) isolation was performed using DNeasy Blood & Tissue Kits (TAKARA) according to the manufacturer’s instruction. Quantitative PCR (qPCR) was performed using THUNDERBIRD SYBR qPCR Mix (TOYOBO Co.) and StepOnePlus Real-Time PCR System (Thermo Fisher Scientific). The temperature profile of real-time PCR was 95°C for 60 s, followed by 40 cycles of 95°C for 10 s and 60°C for 30 s. The following primer sequences were used according to PrimerBank (https://pga.mgh.harvard.edu/primerbank/) (55): mouse beta actin (GenBank Accession: NM_007393): *Actb*_fwd GGCTGTATTCCCCTCCATCG, *Actb*_rev CCAGTTGGTAACAATGCCATGT; mouse C3 (NM_009778): *C3*_fwd CCAGCTCCCCATTAGCTCTG, *C3*_rev GCACTTGCCTCTTTAGGAAGTC; mouse C3aR (NM_009779): *C3ar1*_fwd TCGATGCTGACACCAATTCAA, *C3ar1*_rev TCCCAATAGACAAGTGAGACCAA; mouse C5aR1 (NM_007577): *C5ar1*_fwd CATACCTGCGGATGGCATTCA, *C5ar1*_rev GGAACACCACCGAGTAGATGAT; mouse C1qa (NM_007572): *C1qa*_fwd AAAGGCAATCCAGGCAATATCA, *C1qa*_rev TGGTTCTGGTATGGACTCTCC; mouse complement factor P (properdin) (NM_008823): *Cfp*_fwd TTCACCCAGTATGAGGAGTCC, *Cfp*_rev GCTGACCATTGTGGAGACCT; mouse complement factor B (FB) (NM_008198): *Cfb*_fwd GAGCGCAACTCCAGTGCTT, *Cfb*_rev GAGGGACATAGGTACTCCAGG; mouse complement factor H (FH) (NM_009888): *Cfh*_fwd AGGCTCGTGGTCAGAACAAC, *Cfh*_rev GTTAGACGCCACCCATTTTCC; C4b- binding protein (C4BP) (NM_007576): *C4bp*_fwd ACAAGAGCTGCACATGGGAG, *C4bp*_rev GGCATTGGGTATAGCAGGTGG. The following primer sequences were used for gDNA quantification: mouse beta actin: *Actb*_fwd TGTCTTGATAGTTCGCCATGGA, *Actb*_rev TACAGCCCGGGGAGCATCGT; *T. gondii* B1: *TgB1*_fwd TCTCTCAAGGAGGACTGGCA, *TgB1*_rev GTTTCACCCGGACCGTTTAG.

### ELISA

C5a levels in the cerebral cortex were analyzed by ELISA. Isolated tissues were homogenized in ice-cold RIPA buffer containing protease inhibitor cocktail (Nacalai Tesque, Inc. Kyoto, Japan), centrifuged at 4°C for 15 min, and the supernatants were collected for assay. ELISA was performed using mouse complement C5a ELISA kit according to manufacturer’s instruction (abcam, Cambridge, UK).

### Immunocytochemistry

Microglia-enriched culture with or without pre-treatment with minocycline (20 µM) grown on the glass coverslips were fixed with 4% (w/v) paraformaldehyde for 20 min at room temperature. Cells were permeabilized and blocked using 0.1% (v/v) Triton X-100 (TX100) and 5% (v/v) FBS in Tris-buffered saline (TBS), and incubated overnight at 4°C with primary antibodies: rabbit anti-Glial Fibrillary Acidic Protein (GFAP) (1:1000, Agilent Technologies, Santa Clara, CA USA) and rat anti-CD11b (1:1000, abcam, Cambridge, UK) in TBS containing 0.01% (v/v) TX100. The following secondary antibodies were used: goat anti-rabbit Alexa Fluor 488 and goat anti-rat Alexa Fluor 555, according to the manufacturer’s instructions (Thermo Fisher). Nuclei were stained with DAPI (0.3 µM in TBS). Images were captured using OLYMPUS IX71N-22PH fluorescence microscope with filter sets: U-MWU2 (Ex: 330–385 nm/Em: 420 nm) for blue, U-MNIBA3 (Ex: 470–495 nm/Em 510–550 nm) for green, and U-MWIG3 (Ex: 530–550 nm/Em: 575 nm) for red, equipped with Q Capture Pro 7.0 image analyzer (Media Cybernetics, MD USA).

### Statistical Analysis

Statistical analyses were performed using JMP Pro 13 (SAS Institute Inc., Cary, NC USA). All data were expressed as the mean ± SEM. *P* < 0.05 (by Student’s *t* test, Dunnett’s test, or Tukey’s test) was considered statistically significant.

## Results

### *Toxoplasma* Infection Persistently Increased the mRNA Levels of Alternative Complement Pathway Components and Anaphylatoxin Receptors

**Figure 1:**
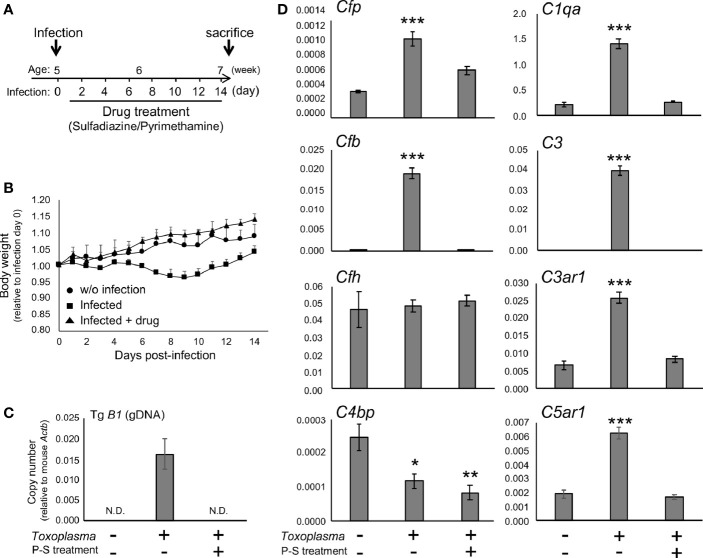
*Toxoplasma* infection induced the expression of alternative complement components and anaphylatoxin receptors in the brain. **(A)** Experimental scheme. C57BL/6J mice were divided into three groups: without infection (n=3), *T. gondii* infection (n=6), and *T. gondii* infection plus drug treatment (Sulfadiazine and pyrimethamine) (n=3). Infection groups received oral inoculation of *T. gondii* (Fukaya, three cysts) on day 0 at the age of 5 weeks, and one group received drug treatment daily from day 1 for 2 weeks until sacrifice. **(B)** Body weight change after *Toxoplasma* inoculation. Data are presented as means ± SEM values relative to body weight at day 0. Filled circle: without infection; filled square: infection; filled triangle: infected with drug treatment. **(C)** *T. gondii* gDNA levels were quantified by qPCR. Values were normalized to mouse β-actin (*Actb*). Data are presented as Means ± SEM. **(D)** mRNA levels for complement components (properdin: *Cfp*, factor B: *Cfb*, factor H: *Cfh*, C4b-binding protein: *C4bp*, C1q: *C1qa*, C3: *C3*, C3aR: *C3ar1*, and C5aR1: *C5ar1*) in the cortex. Data are presented as means ± SEM. *p < 0.05, **p < 0.01 ***p < 0.001 (Dunnett’s test *vs.* control). The representative outcome of two independent experiments are presented.

To determine whether the alternative complement pathway and downstream anaphylatoxin signaling are also affected, mRNA levels for complement components were measured 2 or 8 weeks after oral inoculation. *Toxoplasma* infection induced a transient reduction and gradual recovery of body weight during the first 2 weeks after inoculation (**Figures 1A, B**), consistent with the previous findings showing that systemic parasite dissemination and resolution occur during this period (56, 57). After the initial dissemination, *Toxoplasma* establishes a chronic infection in the brain, which was confirmed by the presence of *Toxoplasma* gDNA in the brain cortex 2 weeks after inoculation (**Figure 1C**). In contrast, the weight gain of mice treated with the combination of sulfadiazine and pyrimethamine, a standard anti-*Toxoplasma* treatment, was comparable to that of control without infection (**Figure 1B**), suggesting that the drug treatment effectively suppressed parasite dissemination. The effectiveness of the drug treatment was also confirmed by the absence of *Toxoplasma* gDNA in the brain tissue of drug-treated mice 2 weeks after infection (**Figure 1C**). Infection-induced alterations in the mRNA expression levels of complement components were assessed by qPCR (**Figure 1D**). Two weeks after infection, C1q (*C1qa*) and C3 (*C3*) expression increased, as suggested previously (42–44). In addition, *Toxoplasma* infection induced a marked up-regulation of properdin (*Cfp*) and CFB (*Cfb*), which was abrogated by anti-*Toxoplasma* drug treatment. CFH (*Cfh*) expression was unaltered, suggesting enhancement specifically of alternative complement pathway activity. Curiously, *Toxoplasma* infection led to a decrease in C4BP (*C4bp*), an inhibitor of the classical and lectin pathways; however, this decrease was not attenuated by the drug treatment. Furthermore, infection induced the expression of C3aR (*C3ar1*) and C5aR1 (*C5ar1*). These results suggest that *Toxoplasma* infection enhances alternative complement pathway activity as well as anaphylatoxin signaling in the brain parenchyma.

**Figure 2:**
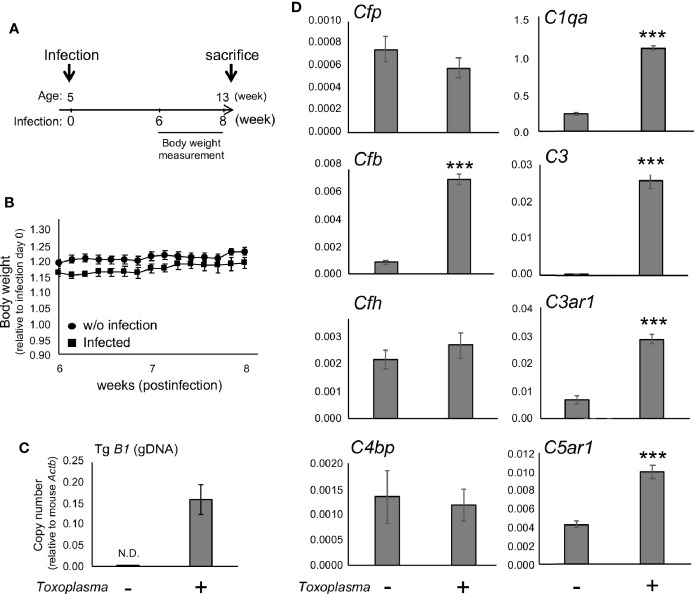
mRNAs for factor B and anaphylatoxin receptors are persistently upregulated after *Toxoplasma* infection. **(A)** C57BL/6J were divided into two groups: with (n=4) or without infection (n=4). The infection group received oral inoculation of *T. gondii* (Fukaya, three cysts) at the age of 5 weeks. **(B)** Body weight was measured before inoculation and from week 6 to week 8 until sacrifice. Body weight change from week 6 to week 8. Data are presented as means ± SEM. **(C)** *T. gondii* gDNA levels were quantified by qPCR. Values were normalized to mouse β-actin (*Actb*). Data are presented as means ± SEM. **(D)** mRNA levels for complement components (properdin: *Cfp*, factor B: *Cfb*, factor H: *Cfh*, C4b-binding protein: *C4bp*, C1q: *C1qa*, C3: *C3*, C3aR: *C3ar1*, and C5aR1: *C5ar1*) in the cortex. Data are presented as means ± SEM. ***p < 0.001 (Student’s t-test *vs.* control). The representative outcome of two independent experiments are presented.

**Figure 3:**
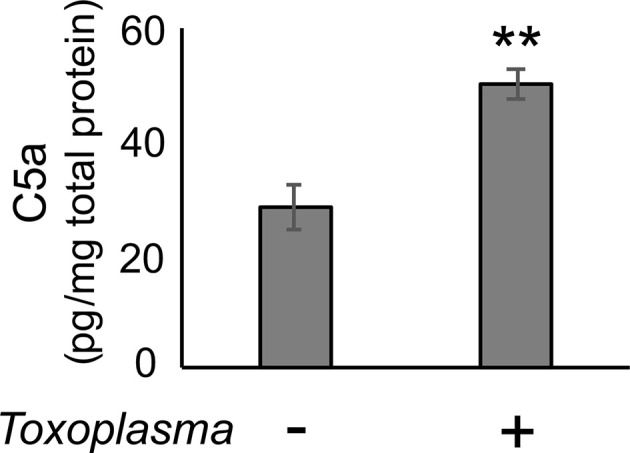
C5a increased in the cerebral cortex after *Toxoplasma* infection. One month after infection, C5a protein levels in the cortical tissue lysates were measured by ELISA. Data are normalized by total protein and presented as means ± SEM (n=4). **p < 0.01 (Student’s t-test *vs.* control). The representative outcome of two independent experiments are presented.

In order to assess whether the observed alterations are sustained, the cortical mRNA levels of complement components were measured 2 months after inoculation (**Figures 2A–C**). *Cfp* levels did not differ between infected and control groups, suggesting that infection-induced upregulation of properdin was transient (**Figure 2D**), and possibly associated with the acute phase of infection. On the other hand, *C1qa*, *C3*, *Cfb*, *C3ar1*, and *C5ar1* expression levels in the cortex remained higher in the infected group compared to the control group (**Figure 2D**), indicating that these factors are persistently upregulated in the brain parenchyma during chronic *Toxoplasma* infection. In addition, C5a protein levels were significantly higher in the cerebral cortex of infected mice, compared to non-infected control (**Figure 3**), supporting that complement cascade is indeed activated, leading to the release of cleavage products.

### *Toxoplasma* Infection Induced the Expression of Complement Components in Glial Cells

**Figure 4:**
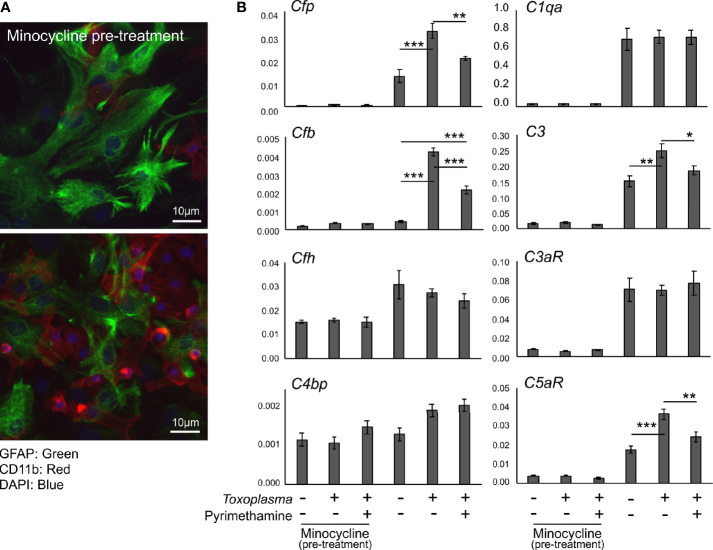
Toxoplasma infection induced the upregulation of alternative complement pathway components and anaphylatoxin C5aR1. **(A)** Primary mixed glial cells prepared from the cortex of postnatal days 1–2 C57BL/6J mice. Astrocytes and microglia were detected by immunofluorescence microscopy, using Glial Fibrillary Acidic Protein (GFAP, green) and CD11b (red) as marker proteins. Cultures were pre-treated either in the presence or absence of minocycline (20 µM) for 5 days prior to *T. gondii* infection (upper panel). Without minocycline pre-treatment, glial culture contained significantly more CD11b positive microglia (lower panel). **(B)** mRNA levels of complement components (*Cfp*, *Cfb*, *Cfh*, *C4bp*, *C1qa*, *C3*, *C3ar1*, and *C5ar1*) in glial cells with or without *T. gondii* infection in the presence or absence of anti-*Toxoplasma* drug pyrimethamine (2 µM). Means ± SEM. *p < 0.05, **p < 0.01 ***p < 0.001 (Tukey’s test).

In order to assess how *Toxoplasma* infection affected the expression of complement components in glial cells, mRNA levels of complement components were determined by qPCR, using murine primary glial cells exposed to *Toxoplasma* for 48 h (**Figure 4**). To address the potential involvement of microglia-astrocyte crosstalk, infection-induced responses were compared between mixed glial cultures, containing both astrocytes and microglia, and cultures pre-treated with minocycline, a microglia inhibitor, prior to infection, which mainly consisted of astrocytes and had significantly fewer microglia (**Figure 4A**). Cell culture without minocycline contained 19.6% of microglia, whereas the percentage of microglia was less than 4.0% after minocycline pre-treatment, according to cell counting (data not shown). Basal expression levels of *Cfp*, *C1qa*, *C3*, *C3ar1*, and *C5ar1* were significantly lower in minocycline pre-treated glia, while *Cfb*, *Cfh* and *C4bp* levels were not affected (**Figure 4B**), suggesting that *Cfp*, *C1qa*, *C3*, *C3ar1*, and *C5ar1* are upregulated in a microglia-dependent manner, either in microglia themselves or in astrocytes dependent on crosstalk with microglia. *Toxoplasma* infection increased mRNA levels of *Cfp*, *Cfb*, *C3*, and *C5ar1*, which was observed only in mixed glial cultures without minocycline pre-treatment (**Figure 4B**). This upregulation was attenuated in the presence of the anti-*Toxoplasma* drug pyrimethamine, confirming that the alterations in the expression pattern were infection-dependent.

## Discussion

The CNS has long been viewed as immunologically privileged. However, it is now evident that glial cells and interactions thereof play important immunological roles in the normal brain as well as in disease (58). This study demonstrated that *Toxoplasma* infection induced alteration in the expression patterns of complement components in the brain and in glial cell cultures. The observation that CFP, CFB, C3, and C5aR1 mRNAs were induced in mixed glial cultures containing both astrocytes and microglia, but not in the cells pre-treated with microglial inhibitor minocycline, suggests that *Toxoplasma* infection enhances alternative complement pathway activity and C5aR1 signaling *via* interactions with microglia. Of note, it has been shown that *Toxoplasma*-infected microglia exhibited hypermotility, suggesting that microglia mediate parasite dissemination in the CNS *via* a ‘Trojan horses’ mechanism (59). It is possible that the hypermotility of *Toxoplasma*-infected microglia also contribute to the propagation of enhanced complement activity in various brain regions. In addition, while *Toxoplasma* infection induced the up-regulation of CFP, CFB, C3, and C5aR1 mRNAs both in the brain and cultured glial cells, C1q and C3aR mRNAs increased only in the brain. These observations suggest that *Toxoplasma* infection directly induces the upregulation of CFP, CFB, C3, and C5aR1 in glial cells whereas the induction of C1q and C3aR requires other cellular components.

Microglia and astrocytes are the major glial populations that regulate homeostasis in the normal brain, and microglia-astrocyte crosstalk determines their functionality (60). Microglia are brain-resident immune cells, monitoring and responding to damage-associated microenvironmental cues and clearing cellular debris (61). In addition, microglia play key roles in synaptic formation, sculpting, and myelination during prenatal and postnatal brain development (62), and directly monitor the functional state of neurons and synaptic activities, and regulate synaptic plasticity in the intact brain throughout the entire lifespan (63). While these cells are essential part of the CNS, their dysfunction and overactivation can lead to neuroinflammation leading to various brain dysfunctions (64, 65). Glial activation has been discussed most frequently in association with increased production of inflammatory cytokines and chemokines (66). Meanwhile, growing evidence suggests significant roles of complement components in microglia-mediated neuropathological events (67–69). Microglia originally arise from yolk sac-primitive macrophages and proliferate gradually during embryogenic development; 95% of the microglial population is established in the early postnatal period (70). Importantly, during the postnatal period, microglia-mediated synaptic elimination and remodeling occur in a complement-dependent manner (71), suggesting that altered microglial complement activity during this period could have detrimental consequences in CNS development. Congenital toxoplasmosis is frequently associated with neurodevelopmental disorders, such as psychomotor retardation, intellectual disability, epilepsy, and autism spectrum disorders (72). In addition, later in life, *Toxoplasma* infection is related to a number of neuropsychiatric disorders, including anxiety, depression, and schizophrenia (72), which are associated with altered glial activity and neuroplasticity (35, 73). Thus, *Toxoplasma*-microglia interaction and infection-induced changes in complement activity could underlie detriments to CNS development and maturation. This study showed that *Toxoplasma* infection upregulated CFP and CFB mRNAs in the brain *via* direct interaction with microglial cells. Activation of the alternative complement pathway has been implicated in various brain disorders, including prion infection (25), suggesting potential contribution of alternative complement pathway activation to the development of cerebral toxoplasmosis. The current study also demonstrated that *Toxoplasma* infection caused sustained upregulation of C5aR1 and C3aR mRNAs in the brain. C5aR1 is primarily expressed on myeloid cells, including microglia (74), and C5aR1 signaling is involved in brain dysfunctions associated with neuroinflammation, such as epilepsy (75) and neurodegenerative disorders (76). Inhibition of C3aR or C5aR1 is frequently associated with neuroprotection against injuries, such as stroke (77–79). Furthermore, murine experimental malaria in pregnancy induced neurocognitive deficits in the offspring *via* C5aR1 signaling, which were independent of *in utero* parasite transfer (24). These data suggest that damage-associated C5aR1 signaling could potentiate neuroinflammation, possibly exacerbating defects in neural development and repair processes. On the other hand, neuronal populations also express complement components (13), and C3aR and C5aR1 are required for neuronal migration during cortical development (13, 17). In addition, C3aR signaling activation enhances neural plasticity after ischemic injury (80), suggesting the roles of C3aR in the protection and repair of the CNS depending on the context. Thus, it is possible that sustained upregulation of C3aR and C5aR1 in the brain after *Toxoplasma* infection is part of the defense against infection-induced damages in the CNS. Similarly, C1q-dependent microglial phagocytosis may be associated with debris-clearance (81) and synaptic reorganization (82), as part of the recovery processes. The significance of neuronal complement activation in the context of *Toxoplasma* infection is another future research direction. Of note, *T. gondii* is classified into virulent (type I) and avirulent (type II and type III) strains. While avirulent *T. gondii* forms tissue cysts, causing long-lasting infection in the brain, virulent *T. gondii* exhibits significantly lower cyst-forming potential (83). It remains to be elucidated whether the complement system responds to *Toxoplasma* infection differently depending on the virulence.

This study demonstrated that *Toxoplasma* infection induced upregulation of alternative complement components and anaphylatoxin receptors, in part mediated *via Toxoplasma*-microglia interaction. The exact nature and significance of the observed phenomenon are unknown; however, it is plausible that infection-induced modulation of complement activity has either detrimental or beneficial effects, determining the fate of cerebral toxoplasmosis. Further research is warranted to evaluate the functional significance.

## Data Availability Statement

The raw data supporting the conclusions of this article will be made available by the authors, without undue reservation.

## Ethics Statement

The animal study was reviewed and approved by Ethics Committee of Chiba University (permit number: A30-149).

## Author Contributions

NS conceived and planned the experiments. NS carried out the experiments with support from KN, KH, YK, and HY. NS wrote the manuscript with support from KN and HY. All authors contributed to the article and approved the submitted version.

## Funding

This work was supported by JSPS KAKENHI (Grant Numbers 19K07839 to NS, 19K07520 and 17KT0124 to KN, and 18KK0454 to YK) and Japan Agency for Medical Research and Development (AMED) (Grant Number JP19fm0208020 and JP20wm0125003 to YK).

## Conflict of Interest

The authors declare that the research was conducted in the absence of any commercial or financial relationships that could be construed as a potential conflict of interest.
